# Mechanical structures of sidewalk plants: Anatomical evaluation

**DOI:** 10.1016/j.sjbs.2023.103647

**Published:** 2023-04-06

**Authors:** T. Al faifi, Abdurrahman S. Masrahi, A. El-Shabasy

**Affiliations:** Biology Department, College of Science, Jazan University, Saudi Arabia

**Keywords:** Sclerenchyma, Collenchyma, Dicot, Monocot, Support, Impact, Adapt, Interference

## Abstract

The mechanical structure of plant tissues has recently attracted a lot of attention. The present study aims to evaluate the importance of collenchymatous and sclerenchymatous tissues in supporting plant species in their harsh environments like road and street plant habitats. Dicots and monocots are classified into different models according to the types of supporting mechanisms. Mass cell percentage and soil analysis are used in this investigation. The tissues are distributed with different percentage masses and arrangements to overcome various severe conditions. Statistical analyses enhance the role of these tissues and clarify their significant values. The gear support mechanism is claimed to be the perfect mechanical method used.

## 1 Introduction

The mechanical design of stems in terrestrial plants has been subjected to much interest and research by most authors. They have emphasized the importance of such tissues as mechanical ones in placing the structural material of land plants. These tissues are collenchyma, vascular bundles, and sclerenchyma which are situated away from the neutral axis and toward the perimeter. These structures have significant implications from both developmental and biomechanical perspectives (Dorota K. and Agata B., 2022). They increase the bending rigidity and strength of aerial stems. They help terrestrial plants to stand up and support the growth of leaves and flowers (Vincent and Jeronimidis, 1991, Ennos, 1993, Speck, 1994, Köhler et al., 2000).

The ability of land plants to react and perceive any mechanical stimuli is crucial for their survival, growth, and reproductive potential. The morphogenetic responses by which plants adapt against different mechanical loads have evolved at different scales from allometric relationships to cellular mechanisms (Cordero et al., 2007) so that the emergence of mechanical tissues was a crucial innovation in the evolution of terrestrial plants and a prerequisite for the appearance of large plant species on different topographic lands (Rowe and Speck, 2004).

The hierarchical organization of mechanical tissues in different plant types enables a fascinating way to adjust the mechanical properties of tissues in response to environmental cues, such as mechanical loading (Speck and Burgert, 2011, Niklas, 2009). These structural adaptations depend on multifactorial dynamical systems as mechanical tissue orientation, stiffness of plant stem, and compositional modification (Reiterer et al., 1999, Köhler and Spatz, 2002, Burgert et al., 2004, Gindl et al., 2004, Burgert and Fratzl, 2009). Besides relative tissue shape, density, and arrangements, cell mass percentage is a crucial factor on the tissue level (Speck and Burgert, 2011).

This study aims to illustrate the mechanical tissue arrangements and percentages in some different dicot and monocot plant species presented in road and street habitats to adapt to such unfavorable conditions.

## 2 Material and methods

### 2.1 Collection of plant material and soil samples

**Plate 1:**
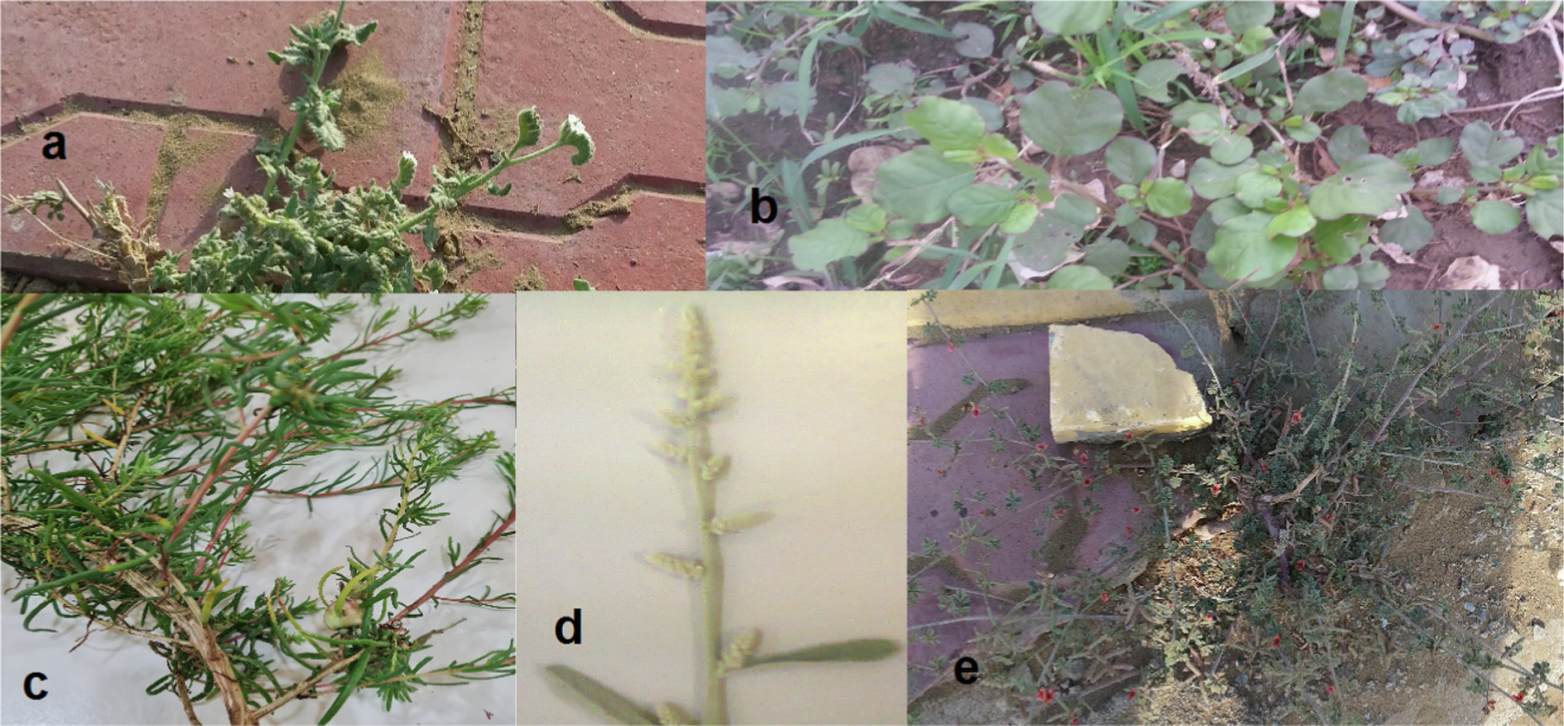
The dicot plants; a. *Heliotropium pterocarpum,* b. *Trianthema portulacastrum* L., c. *Suaeda monoica,* d. *Aerva javanica,* e. *Indigofera colutea.*

**Plate 2:**
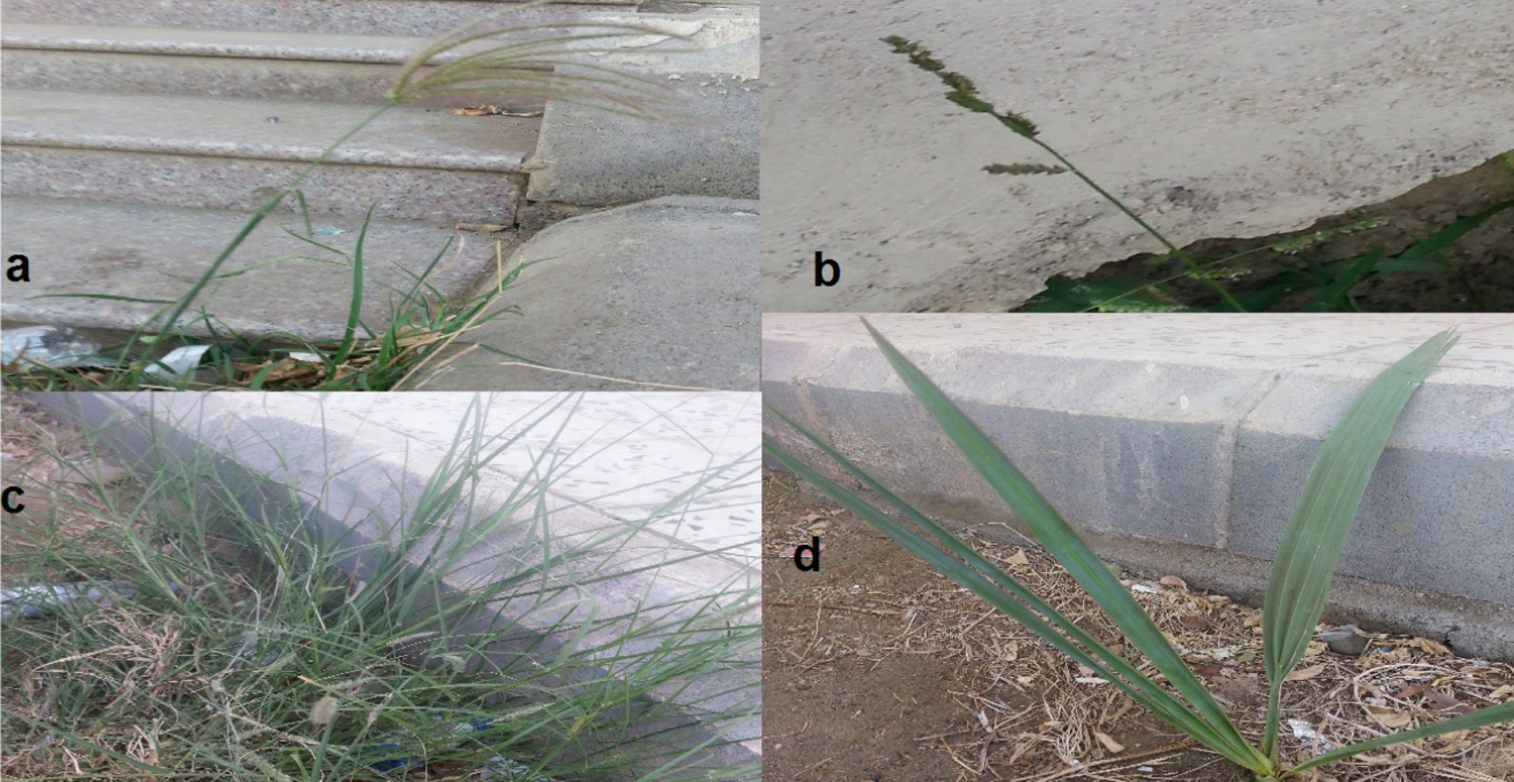
The monocot plants; a. *Chloris gayana*, b. *Echinochloa colona*, c. *Cenchrus ciliaris,* d. *Hyphaene thebaica.*

**Map 1:**
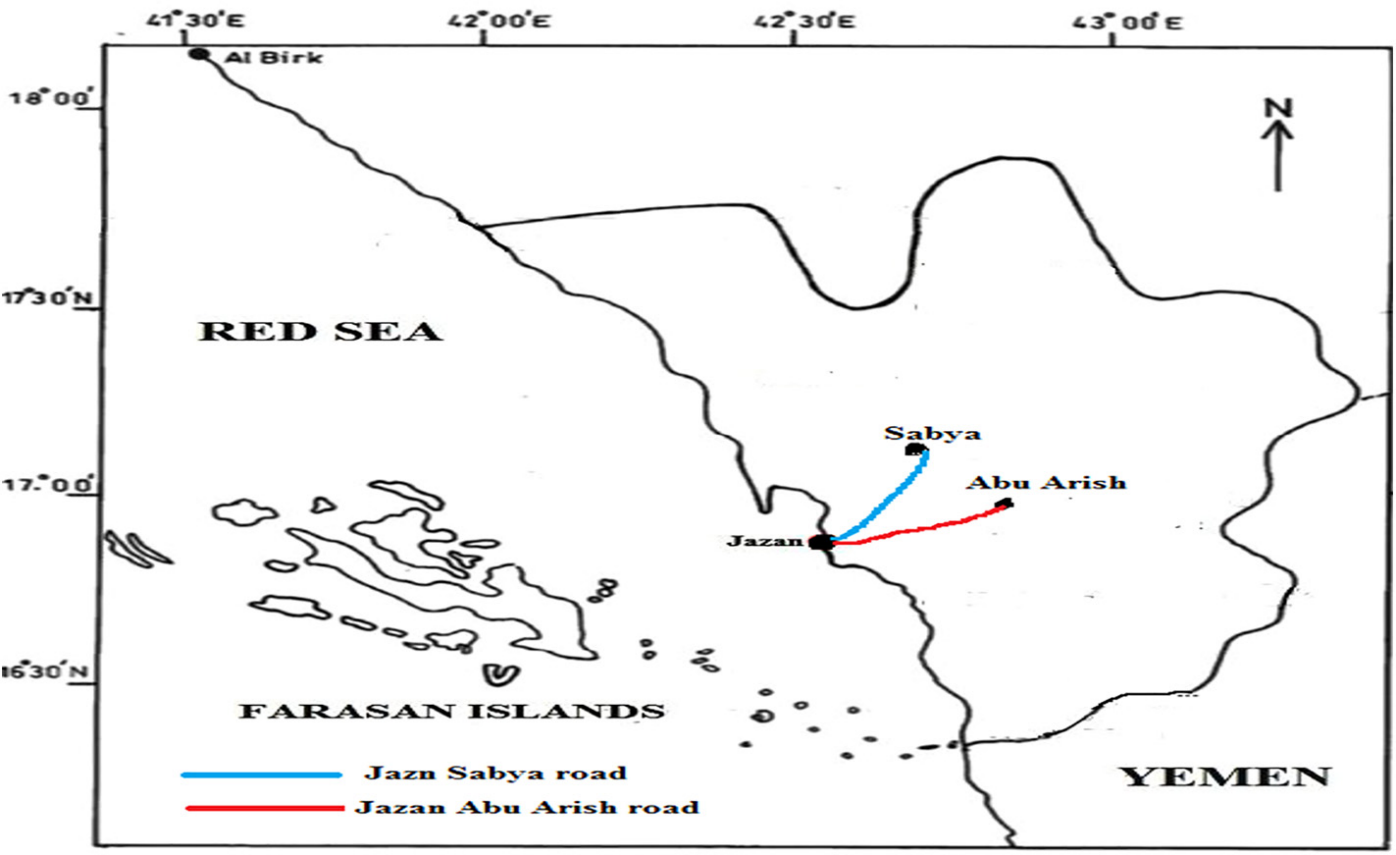
The studied area (E. Atta et al., 2022).

Freshly whole five dicot plant samples; *Heliotropium pterocarpum* (DC.) Steud. & Hochst. *ex* Bunge. (model 1); *Trianthema portulacastrum* L. (model 2), *Suaeda monoica* Forssk. *ex* Gmel. (model 3), *Aerva javanica* (Burm.f.) Juss. *ex* Schult. (model 4) and *Indigofera colutea* (Burm. f.) Merr. (model 5) **(**Plate 1**)** besides freshly whole four monocot plant samples, *Chloris gayana* Kunth (model 1), *Echinochloa colona* (L.) Link (model 2), *Cenchrus ciliaris* L. (model 3), and *Hyphaene thebaica* (L.) Mart. (model 4) were selected for the comparative anatomical stem studies **(**Plate 2**)**. They were collected internally from Jazan province streets and externally from Jazan-Sabya and Jazan-Abu Arish roads, Kingdom of Saudi Arabia in February 2020 **(**Map 1**)** (Coordinates from maps of Saudi Survey Authority). They were identified by the herbarium of the Biology Department, Faculty of Science, Jazan University (JAZUH) **(**Table 1**)**.

**Table 1:** List of studied plant species.

No	Plant species	Plant class	Plant model	Voucher number
1	*Aerva javanica* (Burm.f.) Juss. *ex* Schult.	dicot	4	JAZUH 1304
2	*Cenchrus ciliaris* L.	monocot	3	JAZUH 1305
3	*Chloris gayana* Kunth	monocot	1	JAZUH 1306
4	*Echinochloa colona* (L.) Link	monocot	2	JAZUH 1307
5	*Heliotropium pterocarpum* (DC.) Steud. & Hochst. *ex* Bunge.	dicot	1	JAZUH 1308
6	*Hyphaene thebaica* (L.) Mart.	monocot	4	JAZUH 1309
7	*Indigofera colutea* (Burm. f.) Merr.	dicot	5	JAZUH 1310
8	*Suaeda monoica* Forssk. *ex* Gmel.	dicot	3	JAZUH 1311
9	*Trianthema portulacastrum* L.	dicot	2	JAZUH 1312

The fourth node of the stem samples was sectioned using a razor blade, and the thin slices obtained were kept in water before mounted onto a glass slide where some drops of absolute ethyl alcohol were added for tissue hardening and then two drops of Safranin and light green (1:1; v:v) solutions. The excess stain was washed off with water before adding a drop of glycerine. Slides were covered with coverslips and then ringed with nail lacquer to prevent dehydration. Slides were observed with an Olympus microscope, and photographs were taken with a digitized camera (Nikkon) (Adrian Goodman, 2005, El-Shabasy and Al-Gifri, 2019). The photographs were made to depict the locations of mechanical support tissue, which were differentiated into the parenchyma, vascular tissue, sclerenchyma bundles, and the pith.

### 2.2 Mass cell percentage

The percentage of parenchyma, vessels, collenchyma, fibers, and rays is calculated for each plant stem sample. Since vessels are not exactly circular, the vessel hydraulic diameter D_h_ was calculated from the number of vessels (n) and vessel diameter (d) according to the following equation as Sterck et al., 2008, Poorter et al., 2010:

$$D_{h}={\left[\left(\frac{1}{n}\right)\sum_{i=n}^{n}d^{4}\right]}^{1/4}$$

n = number of vessels and d = vessel diameter.

### 2.3 Soil analysis

The soil samples were also collected along with plant samples from street and road habitats besides natural plant habitats which are perpendicular far away from roads to a distance of 500 m into the sandy plains. All soil samples are collected to a depth of (10–20 cm). Each soil sample was sieved, acid washed, distilled water washed, and stored in a plastic bag at room temperature, being used for analysis. The soil pH was measured using a digital pH meter (with a pH reading meter (Model Jenway PHM 6) in (1:2.5) soil to water ratio, electrical conductivity (EC), total dissolved salts (TDS), water holding capacity (W.H.C.), organic matter were performed (Wilde et al., 1979).

### 2.4 Statistical analysis

D_h_ data for both dicot and monocot models were examined for the descriptive statistical analysis: mean ± SD, variance, standard error of the mean (SEM), 95% confidence interval (CL) of mean, Skewness, kurtosis, Shapiro-Wilk test and *p*-value (Machado et al., 2009). Correlation among vessel of plant samples and regression between a number of vessels and vessel diameters are achieved according to (Shaban, 2005, Tamhane, 2009)**.** P values for significance tests based on degrees of freedom were determined according to Dutilleul (1993) approach. D_h_ and vessel diameter were subjected to statistical analysis to work out ANOVA to compare means. Standard error was calculated following Steel et al., (1997).

Mass cell percentage was tested for significance using Duncan's range test (Duncan, 1951). This test was to evaluate the differences among tissues. Statistical tests were conducted using SPSS software (ver. 22) for Windows (D. Radwan & A. El-Shabasy, 2020).

## 3 Results

### 3.1 Mechanical models

#### 3.1.1 Dicotyledonous stems

**Plate 3:**
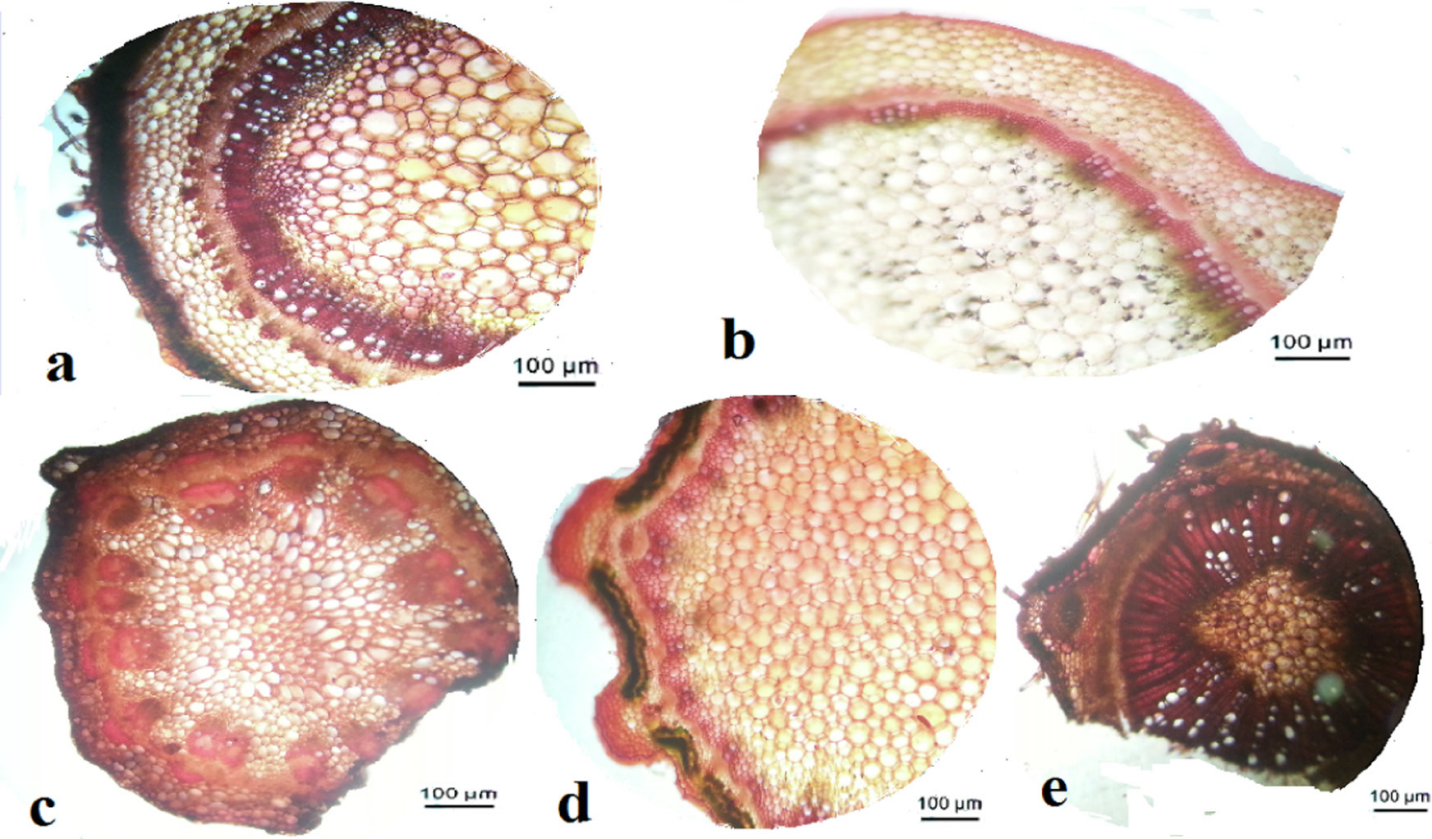
T.S. in dicot stem models a; model 1, b; model 2, c; model 3, d; model 4, e; model 5.

**Fig. 1:**
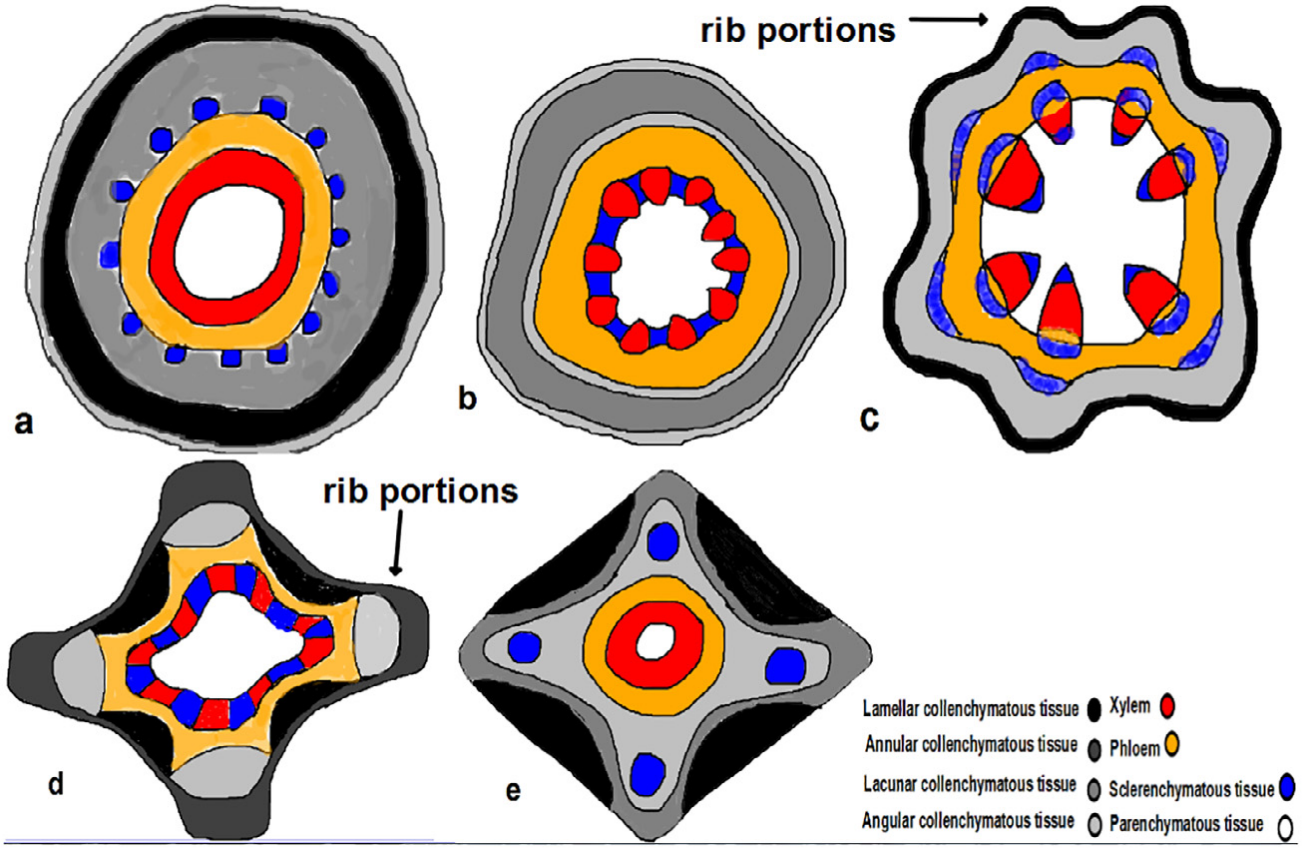
Diagrammatic T.S. in dicot stem models a; model 1, b; model 2, c; model 3, d; model 4, e; model 5.

**(**Plate 3**,**Fig. 1**)** (The first model; Entire section (complete rounded circular section) ex. like *Helitropium*) in this model, the stem is supported peripherally with collenchymatous tissues, which are differentiated into three layers; angular, lamellar, and lacunar. The sclerenchymatous tissues are inner tissues that surround the vascular bundles in the form of horizontal semi-gear structures. Incomplete gear structure is a new mechanical expression used to describe the incomplete annular sclerenchymatous tissue surrounding vascular tissues.

(The second model; Crenate section (entire circular section) ex. *Trianthema*) in this model, the stem is supported peripherally with collenchymatous tissues, which are differentiated into three layers; angular, lacunar, and angular. The sclerenchymatous tissues are fascicular types intersecting with vascular tissues as large vertical dissecting supporting layers.

(The third model; ondulate section (Incomplete rounded circular section) ex. *Suaeda*) in this model, the stem is supported peripherally with collenchymatous tissues, which are differentiated into two layers; lamellar and angular. The sclerenchymatous tissues are annular and are dissected into three layers surrounding vascular tissues; the separated outer layer covers the phloem, the interlayer covers another phloem layer, and the inner one covers the xylem beneath. It looks like doubled vertical dissecting supporting layers.

(The fourth model; ribbed circular section ex. *Aerva*) in this model, the stem is supported peripherally with different collenchymatous tissues. There are two different positions; one is situated at ribs, whose thick annular and angular while the latter is situated between ribs (inter-ribs) whose thin annular and thick lamellar. The sclerenchymatous tissues are presented parallel to the ribs covering the phloem. Other sclerenchymatous tissues are fascicular, with xylem tissue as one small vertical dissecting supporting layer.

(The fifth model; ribbed quadrangular section ex. *Indigofera*). In this model, there is such a complicated mechanical structure. Each rib has different successive layers; lacunar, angular, sclerechymatous, and angular, respectively, but in the inter-ribs regions; there are only three different successive layers; lamellar, lacunar, and angular. It is obvious that the inter-ribs are not supported with sclerenchymatous because it is more or less out of mechanical contact than the ribs.

#### 3.1.2 Monocotyledonous stems

**Plate 4:**
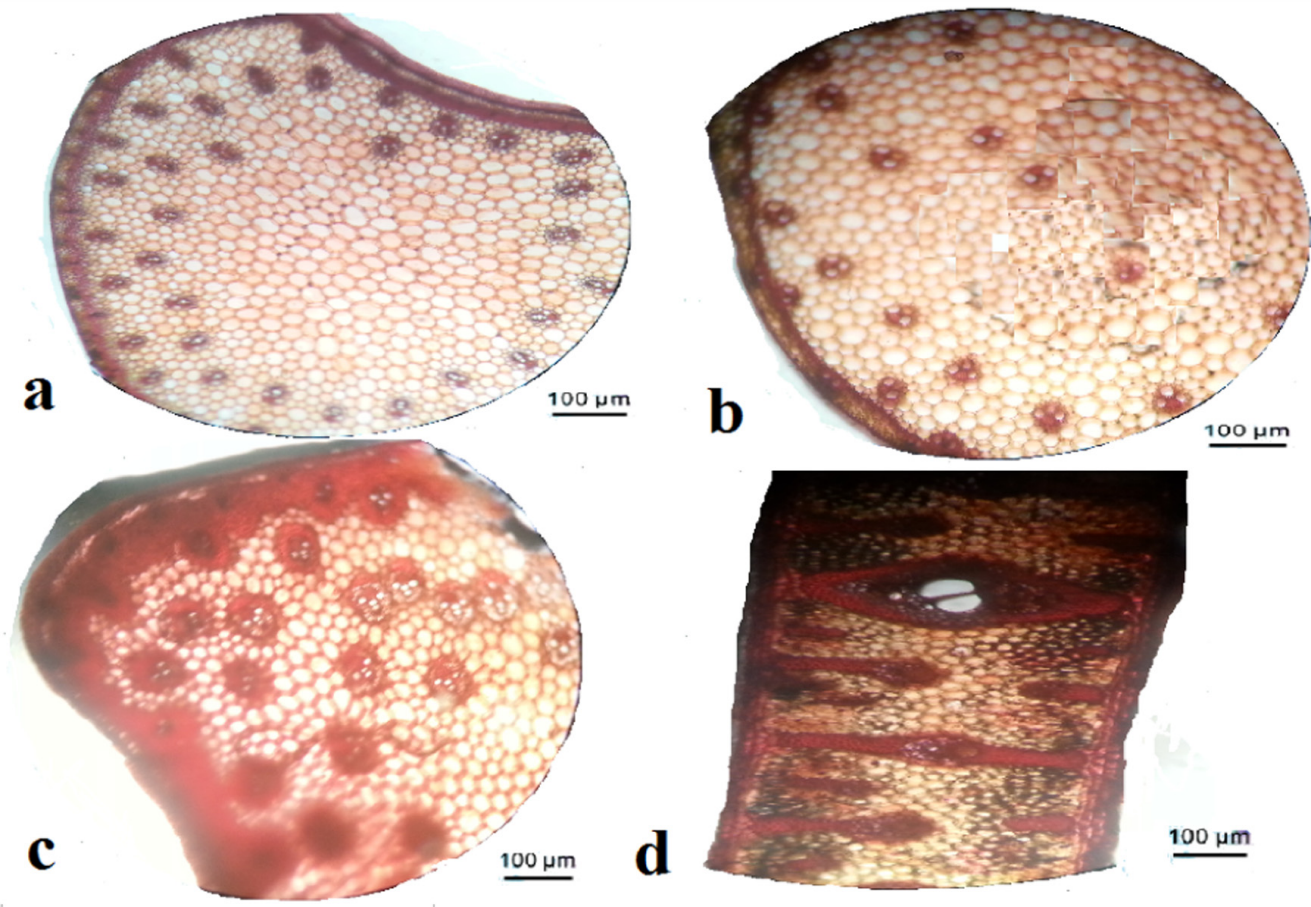
T.S. in monocot stem models a; model 1, b; model 2, c; model 3, d; model 4.

**Fig. 2:**
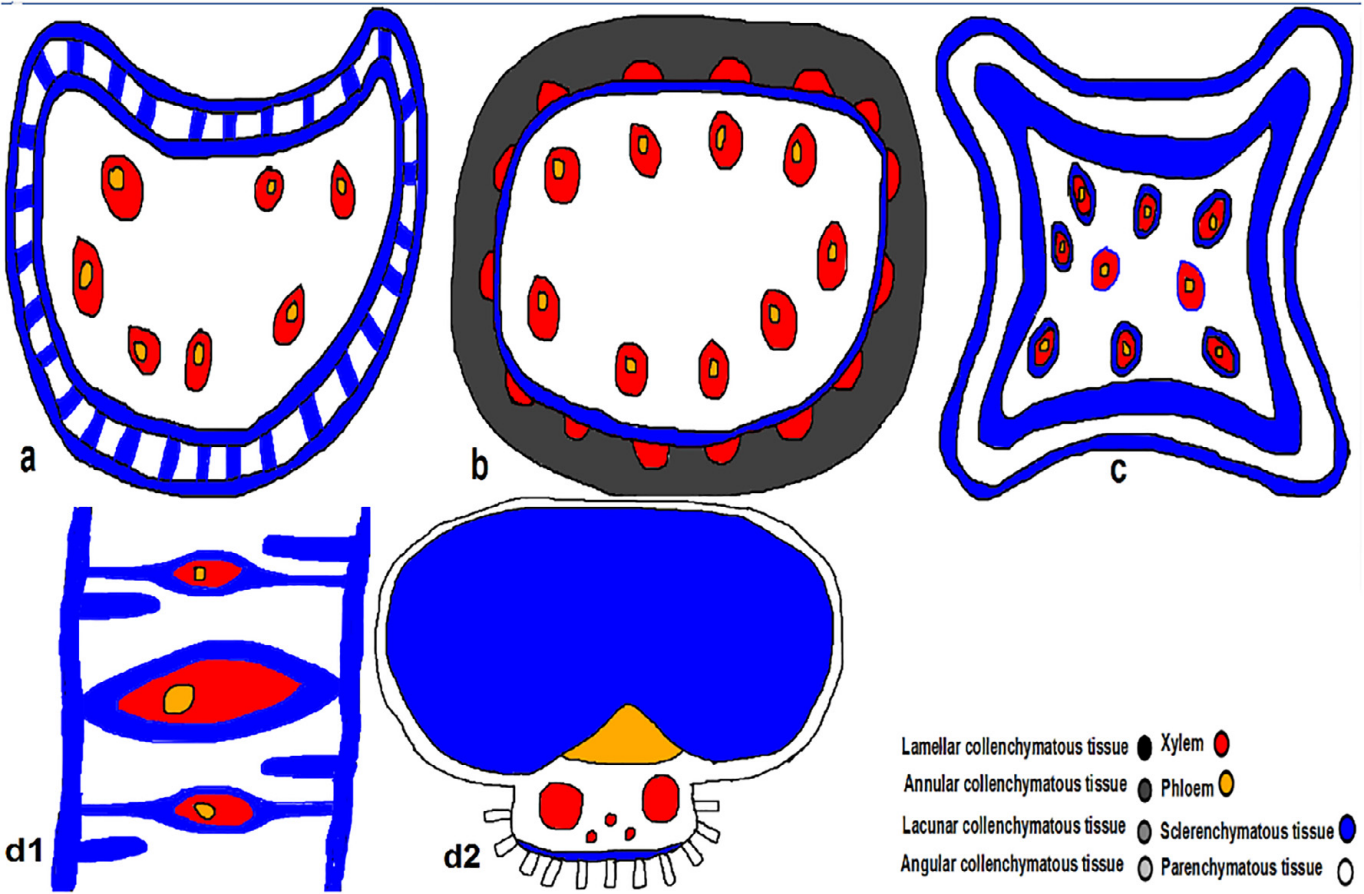
Diagrammatic T.S. in dicot stem models a; model 1, b; model 2, c; model 3, d1; model 4 (leaf section), d2; model 4 (stem section).

**(**Plate 4**,**Fig. 2**)** (The first model; Reniforma section (one curved side of entire circular section) ex. *Chloris*) the perfect gear structure is present where two sclerenchymatous tissues are trapped internally in parallel horizontal strips of the same one.

(The second model; Complanata section (one slightly curved side of the entire circular section) ex. *Echinochloa*) it is the deviation of a perfect mechanical structure where two annular and sclerenchymatous tissues are trapped internally in vascular bundles and other vascular ones outside this structure. Present other vascular bundles in the ground tissue to enhance its cells.

(The third model; Ribbed quadrangular section ex. *Cenchrus*) it is the deviation of a perfect mechanical structure where there are two sclerenchymatous tissues trapped internally in vascular bundles besides there are other vascular ones outside this structure like the previous one, but there is a shield sclerenchymatous layer surrounding each vascular bundle. Its thickness decrease gradually as the vascular bundle involves internally in the ground tissue.

(The fourth model; Palm section (In Palm plants); Ribbed quadrangular section in leaf ex. *Hyphaene*) in this model, the leaf is the first plant part that protrudes outside, facing environmental stresses. In the leaf section, a sclerenchymatous tissue underneath the upper and lower epidermis invades internally, covering each vascular bundle. If the vascular bundle is near to upper epidermis, the sclerenchymatous tissue invades from the upper side and vice versa. The vascular bundle in the middle has invaded sclerenchymatous tissues from both epidermises. The lamellar collenchymatous tissues present under the peripheral sclerenchymatous tissues; otherwise, the angular collenchymatous tissues diffuse the rest of the section.

In the stem section, according to (Romain, 2013), the sclerenchymatous tissues cover the vascular bundles from the upper and lower sides. On the upper side, there is a large mass of sclerenchymatous tissues above the phloem. In contrast, on the lower side, there are microscopic sclerenchymatous tissues beneath the xylem, where there is perivascular parenchyma between the sclerenchymatous tissues and the xylem.

### 3.2 Mass cell percentage

In Dicotyledonous stems, D_h_ and No. vessels are high in the first model while it is medium in the other three models; the second, the fourth and the fifth. The vessel diameter is high in the fifth model, while it is medium in such other three ones; the first, the second and the fourth. The third model has the lowest D_h_ and vessel diameter values **(**Table 2, Table 3**)**.

**Table 2:** No. vessels and diameter of dicot models.

Dicot model	Number of vessel s (n)	Vessel diameter (d)
The first	44 ± 2.5	23.33 ± 6.45 µ
The second	25.4 ± 1.3	18.13 ± 2.30 µ
The third	16 ± 2.5	12.93 ± 1.69 µ
The fourth	24 ± 2.5	19.73 ± 0.58 µ
The fifth	24 ± 2.5	25.6 ± 0.58 µ

**Table 3:** D_h_ analysis of dicot models.

Dicot model	Mean ± SD	Variance	Standard error of mean (SEM)	95% confidence interval (CI) of mean	Skewness	Kurtosis	Shapiro-Wilk test	*p*-value
The first	29.56 ± 1.19	1.42	0.53	28.08–31.04	0.01	−1.94	0.96	0.81
The second	17.18 ± 1.22	1.49	0.55	15.66–18.69	0.01	−2.75	0.89	0.36
The third	10.22 ± 1.68	2.81	0.75	8.14–12.3	0.03	−2.22	0.94	0.66
The fourth	15.14 ± 1.33	1.76	0.59	13.49–16.78	0.61	−1.16	0.91	0.46
The fifth	14.50 ± 1.42	2.019	0.63	12.73–16.26	0.86	0.493	0.94	0.67

The second model has the highest parenchyma percentage but the lowest vessel percentage. In contrast, the fifth model has the lowest and highest parenchyma and vessel percentage respectively. The collenchyma percentage is more or less the same among dicot models, but it is the lowest in the second model. The sclerenchymatous tissues (fibers) percentage is more or less the same among dicot models except for the third model which has the highest value. Ray is present only in the third and fourth models **(**Table 4**) (**Figure 3**)**.

**Table 4:** Tissue percentages of dicot models.

Dicot model	Paranchyma %	Vessel %	Collenchyma %	Fiber %	Ray %
The first	58.46 ± 0.198	18.95% ± 0.07	13.33%0.18	9.26% ± 0.05	0
The second	84.66% ± 0.042	6.56% ± 0.09	2.22% ± 0.10	6.56% ± 0.08	0
The third	46.29% ± 0.06	24.12% ± 0.08	8.71% ± 0.06	17.65% ± 0.06	3.23% ± 0.05
The fourth	57.14 ± 0.16	20.13% ± 0.12	13.31% ± 0.17	8.44% ± 0.18	0.98% ± 0.04
The fifth	26.44% ± 0.14	50.89% ± 0.14	13.39% ± 0.16	9.28% ± 0.06	0

In monocots, both the first & third models have the highest D_h_ and the lowest vessel diameter values, respectively. On the other hand, both the third & fourth models have the highest No. of vessels and diameter vessel values, respectively. All of the second & fourth models have low D_h_ values. Only the fourth model has the lowest number of vessels value **(**Tables 5, 6**)**.

All monocot models have more or less parenchyma percentage except the third model, which has the lowest value. The first & fourth models have the highest vessel percentage values, while the other models have the lowest ones. On the contrary, the second & the third models have the highest collenchyma percentage while the others have the lowest values. The highest number of sclerenchyma tissues (fiber) % was present only in the third model. No ray appeared in the monocot models **(**Table 7**) (**Figure 3**)**.

### 3.3 Soil analysis

The porosity, pH, and W.H.C. values are nearly identical for both plant habitats (road & street habitat and wild one). The soil of the platform has the highest sand %, TDS, and conductivity, while the soil of the wild habitat has the highest organic matter, clay %, and moisture content. Coarse and medium sand particles are higher in the soil habitat than in platform one, but fine and very fine sand particles are high and vice versa **(**Tables 8, 9**)**.

### 3.4 Statistical analysis

The regression curve is drawn between the two types of soils **(**Figure 4**)**.

In contrast, all Dicot plant samples have positive Skewness D_h_ values, and all Monocot plant samples have negative skewness ones except the third model. All D_h_ Kurtosis have negative values except the fifth model of dicots. The Shapiro-Wilk test values are near to one where the second model of monocot has got it **(**Tables 3, 6**)**.

The analysis of variance for various quantitative criteria of mechanical tissues by ANOVA test showed highly significant differences for all the indices like D_h_ versus vessel % parameters **(**Table 10**)**. Correlation analysis showed that *Echinochloae* and *Trianthema* had a highly positive significant correlation with *Heliotropium*. However, further inspection revealed that they were better predictors of mean *Heliotropium* than the other indices **(**Table 11**)**. Regression describes the co-variation between several vessel and vessel diameter variables **(**Table 12**)**. Simple Linear Regression curves indicate the significant relationships between vessel % versus other tissues %. Collenchyma % and ray % were highly favorable and weak positively correlated, respectively, while fibers % and parenchyma % were weak and highly negatively correlated, respectively **(**Figure 5**)**.

## 4 Discussion

Anatomy of tolerant dicots and monocots differs substantially according to their habitats (Stefan P. and Wilhelm B.2012). The platform habitat faces such a harsh environment due to human interference. Plants face many physical stress and strain, such as living (human & animal impacts) and non-living (vehicle impact) pressure, high wind speed and wind-carrying obstructed objects. These physical forces influence the soil's moisture content and raising the soil's wilting point. The famous erosion factor is wind which carries soil particles from one place to another. That is why there is an increment in the platform habitat's fine and very fine sand particles. Despite the TDS and Conductivity of the platform habitat being higher than the wild one, the platform habitat is distinguished by vehicle fumes that enrich the soil with heavy metals and harmful particles. Biological stress is considered further stress, represented by the low content of organic matter due to severe environmental conditions. High wind speed increases the sand percentage in the platform habitat. The regression curve confirms the highly significant relationship between the two habitats, which means that they are the same in the past without human intervention. From all of that, not all plants can withstand these hard stresses. These plants should have special strength with different support mechanisms to live within these habitats. A similar finding was reported for (Nitta, 2006) who exhibited a high degree of anatomical alterations in epilithic species compared to whose are often times exposed to extreme environmental conditions.

Dicot plants; have different models to overcome the difficulties and fulfill their requirements. There are different strategies that the plants can follow to give up these unfavorable conditions according to the plant's habit and type. The mechanical gear structure is considered the perfect support the plant can use in these conditions. It presents in model 1 as semi gear structure because the section is circular and has low mechanical support. In other dicot models (the second and the third), it is not present because they have curvature in the section, *i.e.*, the curvature is a type of support (due to low stress action on surface). Instead of gear structure, the sclerenchymatous tissues present as one or double layers, whether small or large, according to the degree of curvature in the models 2 &3. Although model 3 has an amount of rays which is not present in model 2, and more vessel percentage and more supporting tissues percentage than model 2, model 2 has more D_h_ than model 3. The study assumed that model 2 has a more effective vessel than model 3.

On the other hand, models 4 and 5 have the perfect curvature in the sections. The rib portions have more facing edges against any other side of the section, so the rib portions are characterized by definite arrangements of supporting tissues. Due to the high degree of curvature in model 5, it loses the fascinating sclerenchymatous tissue present in model 4. The percentage of supporting tissues is nearly the same between models 4 and 5 besides the D_h_ which is also nearly the same, but model 5 has more vessel percentage. Model 5 is the perfect support model.

In monocot models, models 1 & 2 have less curvature than the others. So, the perfect gear structure is present in model 1, while its deviation is present in model 2. From Table 2, Table 3, the percentage of supporting tissues in model 1 is less than in model 2. This implies that the gear structure is the perfect supporting structure that compensates for the low percentage of supporting tissues. On the other side, model 3 is better than the previous ones because it has more curvature in the section beside the deviated gear structure. Finally, model 4 is regarded as the super-perfect supporting one because the supporting mechanisms are distributed in the leaf and stem. It has the most different mechanical method, which depends on protruding leaves and then completing with stem growth. It agrees with Noreen et al., (2017) who studied the anatomical adaptations of *Typha domingensis* Pers. ecotypes for salinity tolerance.

D_h_ has the highest values in monocot models than dicot ones. It refers to the nature of the stem, which differs from the dicot one. Most monocot models have the minus degree of skewness in D_h_, which means there are incompatible values between No. of vessels and vessel diameter. It reflects that these models do not depend mainly on the vessel for support.

On the other hand, the minus degree of kurtosis of D_h_ shows that the data is more homogenous. In contrast, the only positive one for the fifth model of dicots shows that it has replicated values due to the stable support structure. The Shapiro-Wilk test has data near the right one to illustrate that the replica of each model increases gradually. D_h_ values are in agreement with the results of previous study (Karen et al., 2007) who reported that Anatomical changes may affect the mechanical properties of the tissue even in cases where there is no apparent trade-off between hydraulic and mechanical parameters.

The combination of the *p*-value and F-value of D_h_ and vessel percentage from one side and the number of vessels and vessel diameter from another means that the corresponding variables would be highly significant. Correlation analysis showed that model 2 of a monocot and model 2 of a dicot had a highly positive significant correlation with model 1 of dicot. They were better predictors of mean model 1 of a dicot than the other indices. Further, inspection revealed that they have more or less the same effective potential support tissues due to deviated gear structure. In contrast, model 2 of the dicot and model 3 of the monocot have a highly negative significant correlation with model 2 of monocot, which reflects that they have the most different support mechanisms.

Simple Linear Regression of the significant relationships for the vessels versus other plant tissues indicated that vessels are compatible with collenchyma tissues with parallel relation while the sclerenchyma tissues are in reversible attributed relation with vessel corresponding to vessel %. It resembles to (Nada et al., 2003) who stated that the plant gets its strength from sclerenchyma fibers pervading the whole length of the stem. The mechanical strength may arise from the tangential and normal components of the cell-generated stresses (Serrano, R. et al. 2019).

## 5 Conclusion

The plant habit undergoes different mechanisms to adapt to its habitat. The gear support mechanism is regarded as the perfect mechanical method to overcome a variety of stresses that are increasing nowadays. Human interference causes alteration of plant performance positively or negatively, which may not be able to withstand in the future.

## Declaration of Competing Interest

The authors declare that they have no known competing financial interests or personal relationships that could have appeared to influence the work reported in this paper.
