# Supplementary material for: Mechanical structures of sidewalk plants: Anatomical evaluation
Source: Saudi J Biol Sci. 2023 Apr 6;30(6):103647. doi: 10.1016/j.sjbs.2023.103647 (PMC10173764; doi:10.1016/j.sjbs.2023.103647)
Supplement: Supplementary data 1 [file mmc1.docx]

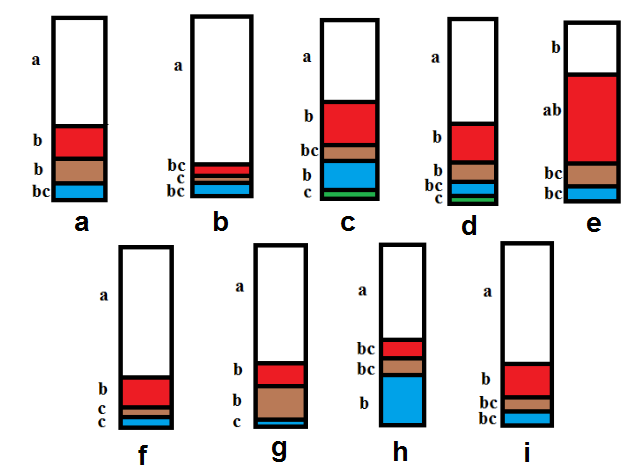
Rest figures in a supplementary section

**Fig.** **3**. Tissue percentages of all models; a-e; 1-5 dicot models, f-i; 1-4 monocot models.


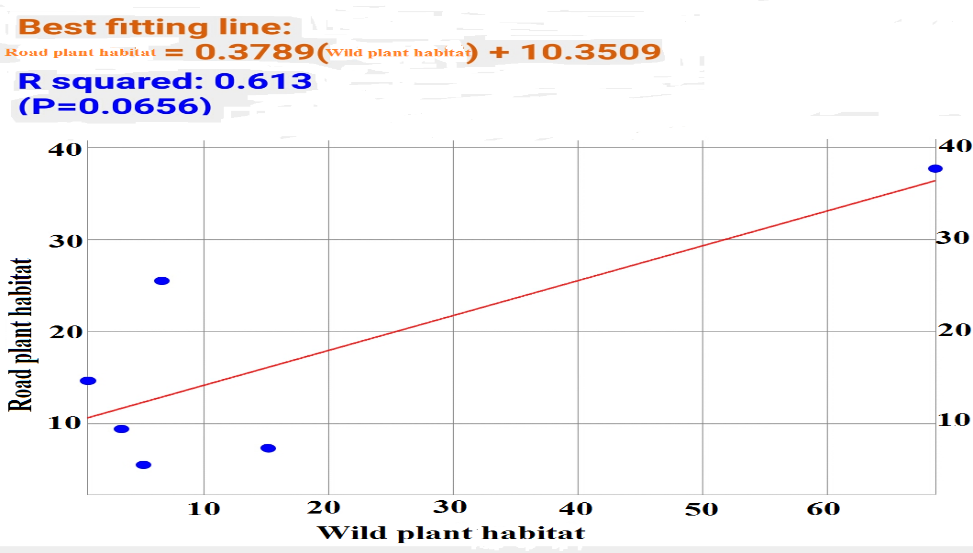


**Fig. 4**. Simple Linear Regression of the significant relationships between two types of soil.


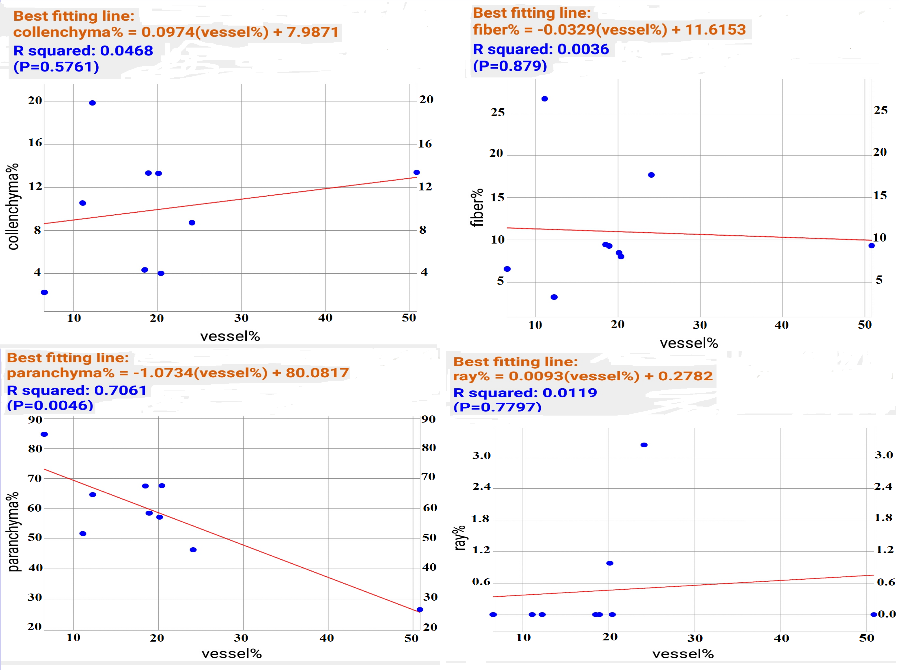


**Fig.** **5**. Simple Linear Regression of the significant relationships between vessel % versus other tissues %.
