# Supplementary material for: Mechanical structures of sidewalk plants: Anatomical evaluation
Source: Saudi J Biol Sci. 2023 Apr 6;30(6):103647. doi: 10.1016/j.sjbs.2023.103647 (PMC10173764; doi:10.1016/j.sjbs.2023.103647)
Supplement: Supplementary data 2 [file mmc2.docx]

Rest tables in a supplementary section

**Table** **5.** No. vessels and diameter of monocot models.

| Monocot model | Number of vessel s (n) | Vessel diameter (d) |
| --- | --- | --- |
| The first | 46±2.5 | 10.27±6.58 µ |
| The second | 38±2.5 | 18.4±5.91 µ |
| The third | 51±2.5 | 12.79±3.42 µ |
| The fourth | 26±2.5 | 37.47±196.53 µ |

**Table** **6**. D_h_ analysis of monocot models.

| Monocot model | Mean±SD | Variance | Standard error of mean (SEM) | 95% confidence interval (CI) of mean | Skewness | Kurtosis | Shapiro-Wilk test | *P*-value |
| --- | --- | --- | --- | --- | --- | --- | --- | --- |
| The first | 31.17±1.06 | 1.11 | 0.47 | 29.86-32.49 | -0.01 | -1.17 | 0.99 | 0.97 |
| The second | 25.68±1.09 | 1.18 | 0.49 | 24.33-27.02 | -0.15 | -0.47 | 1.00 | 1.00 |
| The third | 34.51±1.06 | 1.12 | 0.47 | 33.20-35.82 | 0.01 | -1.21 | 0.99 | 0.96 |
| The fourth | 27.64±13.25 | 175.54 | 5.92 | 11.19-44.09 | -0.01 | -1.407 | 0.97 | 0.87 |

**Table** **7**. Tissue percentages of dicot models.

| Monocot model | Paranchyma % | vessel% | Collenchyma% | Fiber% | Ray% |
| --- | --- | --- | --- | --- | --- |
| The first | 67.47%±0.36 | 18.51%±0.12 | 4.31%±0.02 | 9.44%±0.06 | 0 |
| The second | 64.62%±0.12 | 12.28%±0.03 | 19.88%±0.08 | 3.22%±0.06 | 0 |
| The third | 51.67%±0.36 | 11.11%±0.08 | 10.55%±0.07 | 26.67%±0.06 | 0 |
| The fourth | 67.60%±0.30 | 20.4%±0.11 | 4%±0.35 | 8%±0.07 | 0 |

**Table** **8**. Physical and chemical parameters of both soils.

| **Physical soil parameter** | **Soil of platform** | **Soil habitat** |
| --- | --- | --- |
| **Porosity** | 80% | 79.5% |
| **Organic matter** | 2.59% | 6.15% |
| **% Sand** | 86.2% | 69.23% |
| **% Clay & Silt** | 13.8% | 30.77% |
| **TDS** | 323 ppm | 192 ppm |
| **Conductivity** | 646 µs/cm | 384 µs/cm |
| **pH** | 7.49 | 7.35 |
| **W.H.C** | 49% | 46% |
| **Moisture content%** | 0.84±0.004 | 7.47±0.013 |

**Table** **9**. Particle size analysis of both soils.

| **Soil texture** | **Soil of platform (100g)** | **Soil habitat (100g)** |
| --- | --- | --- |
| **Gravel and very coarse sand particles (4000µm)** | 4.50±5.41 | 3.20±4.89 |
| **Coarse Sand particles (2000µm)** | 6.30±0.61 | 13.23±0.52 |
| **Medium sand particles (500µm)** | 36.70±15.30 | 47.70±16.41 |
| **Fine sand particles (250µm)** | 24.47±0.81 | 4.70±6.24 |
| **Very fine sand particles (125µm)** | 14.23±1.00 | 0.40±0.22 |
| **Silt and clay particles (<125µm)** | 13.80±13.81 | 30.77±6.80 |

**Table** **10**. ANOVA: Two-Factor Without Replication between D_h_ and vessel percentage.

|  |  |  |  |  |  |  |
| --- | --- | --- | --- | --- | --- | --- |
| SUMMARY | Count | Sum | Average | Variance |  |  |
| *Avena* | 2 | 35.27 | 17.635 | 12.45005 |  |  |
| *Cenchrus* | 2 | 45.62 | 22.81 | 273.78 |  |  |
| *Chloris* | 2 | 49.68 | 24.84 | 80.1378 |  |  |
| *Echinochloa* | 2 | 37.96 | 18.98 | 89.78 |  |  |
| *Heliotropium* | 2 | 48.51 | 24.255 | 56.28605 |  |  |
| *Hyphane* | 2 | 48.04 | 24.02 | 26.2088 |  |  |
| *Indigofera* | 2 | 65.39 | 32.695 | 662.1161 |  |  |
| *Suaeda* | 2 | 34.34 | 17.17 | 96.605 |  |  |
| *Trianthema* | 2 | 23.74 | 11.87 | 56.3922 |  |  |
|  |  |  |  |  |  |  |
| D_h_ mean | 9 | 205.6 | 22.84444 | 75.2484 |  |  |
| Vessel percentage | 9 | 182.95 | 20.32778 | 161.6204 |  |  |
|  |  |  |  |  |  |  |
|  |  |  |  |  |  |  |
| ANOVA |  |  |  |  |  |  |
| Source of Variation | *SS* | *df* | *MS* | *F* | *P-value* | *F crit* |
| Rows | 569.6957 | 8 | 71.21196 | 0.429876 | 0.873134 | 3.438101 |
| Columns | 28.50125 | 1 | 28.50125 | 0.17205 | 0.689196 | 5.317655 |
| Error | 1325.255 | 8 | 165.6568 |  |  |  |
|  |  |  |  |  |  |  |
| Total | 1923.452 | 17 |  |  |  |  |

**Table** **11**. Correlation among vessel plant species

|  | *Avena* | *Cenchrus* | *Chloris* | *Echinochloa* | *Heliotropium* | *Hyphane* | *Indigofera* | *Suaeda* | *Trianthema* |
| --- | --- | --- | --- | --- | --- | --- | --- | --- | --- |
| *Avena* | 1 |  |  |  |  |  |  |  |  |
| *Cenchrus* | 0.436114 | 1 |  |  |  |  |  |  |  |
| *Chloris* | 0.234415 | 0.232231 | 1 |  |  |  |  |  |  |
| *Echinochloa* | -0.19518 | -0.75225 | 0.076982 | 1 |  |  |  |  |  |
| *Heliotropium* | -0.64688 | -0.61316 | -0.07857 | 0.820695 | 1 |  |  |  |  |
| *Hyphane* | -0.65616 | 0.303309 | -0.16649 | -0.17108 | 0.40308 | 1 |  |  |  |
| *Indigofera* | 0.173716 | 0.719107 | 0.783276 | -0.48151 | -0.37224 | 0.234129 | 1 |  |  |
| *Suaeda* | 0.572726 | 0.491841 | -0.54919 | -0.37917 | -0.44537 | -0.04872 | -0.23191 | 1 |  |
| *Trianthema* | 0.783678 | 0.743321 | -0.03545 | -0.74246 | -0.91664 | -0.30192 | 0.299536 | 0.73137 | 1 |

**Table** **12.** Regression between the number of vessel and vessel diameter

| SUMMARY OUTPUT | |  |  |  |  |  |  |  |
| --- | --- | --- | --- | --- | --- | --- | --- | --- |
|  |  |  |  |  |  |  |  |  |
| *Regression Statistics* | |  |  |  |  |  |  |  |
| Multiple R | 0.292961 |  |  |  |  |  |  |  |
| R Square | 0.085826 |  |  |  |  |  |  |  |
| Adjusted R Square | 0.064566 |  |  |  |  |  |  |  |
| Standard Error | 11.32032 |  |  |  |  |  |  |  |
| Observations | 45 |  |  |  |  |  |  |  |
|  |  |  |  |  |  |  |  |  |
| ANOVA |  |  |  |  |  |  |  |  |
|  | *df* | *SS* | *MS* | *F* | *Significance F* |  |  |  |
| Regression | 1 | 517.3398 | 517.3398 | 4.036996 | 0.050817 |  |  |  |
| Residual | 43 | 5510.438 | 128.1497 |  |  |  |  |  |
| Total | 44 | 6027.778 |  |  |  |  |  |  |
|  |  |  |  |  |  |  |  |  |
|  | *Coefficients* | *Standard Error* | *t Stat* | *P-value* | *Lower 95%* | *Upper 95%* | *Lower 95.0%* | *Upper 95.0%* |
| Intercept | 40.25519 | 4.086271 | 9.851327 | 1.36E-12 | 32.01444 | 48.49594 | 32.01444 | 48.49594 |
| X Variable 1 | -0.37666 | 0.187464 | -2.00923 | 0.050817 | -0.75472 | 0.001399 | -0.75472 | 0.001399 |
